# Tertiary lymphoid structures in lung adenocarcinoma: characteristics and related factors

**DOI:** 10.1002/cam4.4796

**Published:** 2022-07-08

**Authors:** Fangping Ren, Mei Xie, Jie Gao, Chongchong Wu, Yang Xu, Xuelei Zang, Xidong Ma, Hui Deng, Jialin Song, Aiben Huang, Li Pang, Jin Qian, Zhaofeng Yu, Guanglei Zhuang, Sanhong Liu, Lei Pan, Xinying Xue

**Affiliations:** ^1^ Department of Respiratory and Critical Care, Beijing Shijitan Hospital Capital Medical University Beijing P.R. China; ^2^ Department of Respiratory and Critical Care the Chinese PLA General Hospital Beijing P.R. China; ^3^ Department of Pathology the Chinese PLA General Hospital Beijing P.R. China; ^4^ Department of Radiology the Chinese PLA General Hospital Beijing P.R. China; ^5^ Center of Clinical Laboratory Medicine, the first Medical Centre Chinese PLA General Hospital Beijing P.R. China; ^6^ Department of Respiratory Medicine Weifang Medical university Weifang People’s Republic of China; ^7^ School of Medicine Peking University Beijing P.R. China; ^8^ Institute of Interdisciplinary Integrative Medicine Research Shanghai University of Traditional Chinese Medicine Shanghai P.R. China; ^9^ Shanghai Key Laboratory of Gynecologic Oncology, Renji Hospital, School of Medicine Shanghai JiaoTong University Shanghai P.R. China

**Keywords:** part‐solid nodule, pure ground‐glass nodule, solid nodule lung adenocarcinoma, solitary pulmonary nodule, tertiary lymphoid structure

## Abstract

**Objective:**

Tertiary lymphoid structures (TLSs) are found in a variety of malignancies and affect the growth of tumors, but few studies have addressed their role in lung adenocarcinoma (LAC). We aimed to evaluate clinical features associated with TLSs in patients with LAC.

**Methods and Materials:**

A collection of resected pulmonary nodules in patients with LAC was retrospectively analyzed. TLSs were quantified by their number per square millimeter tumor area (density) and by the degree of lymphocyte aggregation (maturity) in each case. The correlation between TLS density and maturity and clinical features was calculated.

**Results:**

A total of 243 patients were selected, of whom 219 exhibited TLSs. The occurrence of TLSs was correlated with computed tomography (CT) features as follows: pure ground‐glass nodules (pGGNs) (*n* = 43) was associated with a lower occurrence rate than part‐solid nodules (PSNs) (*n* = 112) and solid nodules (SNs) were (*n* = 88) (*p* = 0.037). TLS density was correlated with age and CT features. Poisson regression showed higher TLS density in PSNs and SNs than in pGGNs (incidence rate ratio [IRR]: 3.137; 95% confidence interval [CI]: 1.35–7.27; *p* = 0.008 and IRR: 2.44; 95% CI: 1.02–5.85; *p* = 0.046, respectively). In addition, TLS density was higher in patients aged under 60 years than in those aged over 60 years (IRR: 0.605; 95% CI: 0.4–0.92; *p* = 0.018). The maturity of TLSs was higher in patients with higher tumor stages (*p* = 0.026).

**Conclusions:**

We demonstrated distinct profiles of TLSs in early LAC and their correlations with CT features, age, and tumor stages, which could help understand tumor progression and management.

## Graphical abstract

Tertiary lymphoid structures (TLSs) were found in 90.12% of the total patients, with Grade I (66.67%), Grade II (26.48%), and Grade III (6.85%). TLSs were more commonly associated with PSN and SN (OR 0.29, *p* = 0.018). The density of TLSs was higher in PSNs and SNs than in pGGNs (IRR: 3.137; 95% CI: 1.35–7.27; *p* = 0.008 and IRR: 2.44; 95% CI: 1.02–5.85; *p* = 0.046, respectively).

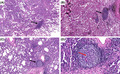

## 1 INTRODUCTION

Tertiary lymphoid structures (TLSs) are ectopic lymphoid formations with highly ordered T and B lymphocyte colonies found in nonlymphoid tissues.^1^, ^2^ Similar to normal lymph nodes, TLSs provide a microenvironment for the recruitment of T cells, activation of B cells, and production of antibodies.^3^, ^4^, ^5^ TLSs were initially found to correlate with favorable clinical outcomes in non‐small cell lung cancer (NSCLC),^6^ and in other similar malignancies, such as gastric, breast, and colorectal cancers.^7^, ^8^, ^9^, ^10^ TLSs have been proven to improve antitumor responses and predict the efficacy of immunotherapy.^11^, ^12^, ^13^, ^14^ The beneficial effect of TLSs on tumors positively correlates with their density and maturity in the tumor area; however, due to their remarkable heterogeneity, no effective method was found to predict TLSs.^13^, ^15^, ^16^, ^17^, ^18^

Although TLSs have been extensively studied in NSCLC, only a handful of studies have been carried out on their role in lung adenocarcinoma (LAC). Furthermore, those studies have mainly focused either on the function of TLSs components, such as protective B cells,^19^ infiltrating T cells,^20^ and immuno‐suppressive regulatory T cells^21^ or on the possible strategies to modulate TLSs.^22^, ^23^ However, whether the clinical features such as age, sex, smoking, tumor stage, or *EGFR* mutational status contribute to TLSs heterogeneity is largely unknown. This lack of knowledge is caused in particular by the lack of information on the relationship between TLSs and lung computed tomography (CT) features, which provide details on tumor tissue in vitro and play an important role in the management of early LAC. The answer to these questions might shed light on the heterogeneity of TLSs, as well as on their role in early LAC pathogenesis and management. Therefore, this study aimed to investigate the clinical characteristics associated with TLS in patients with LAC

## 2 MATERIALS AND METHOD

### 2.1 Subjects

**FIGURE 1:**
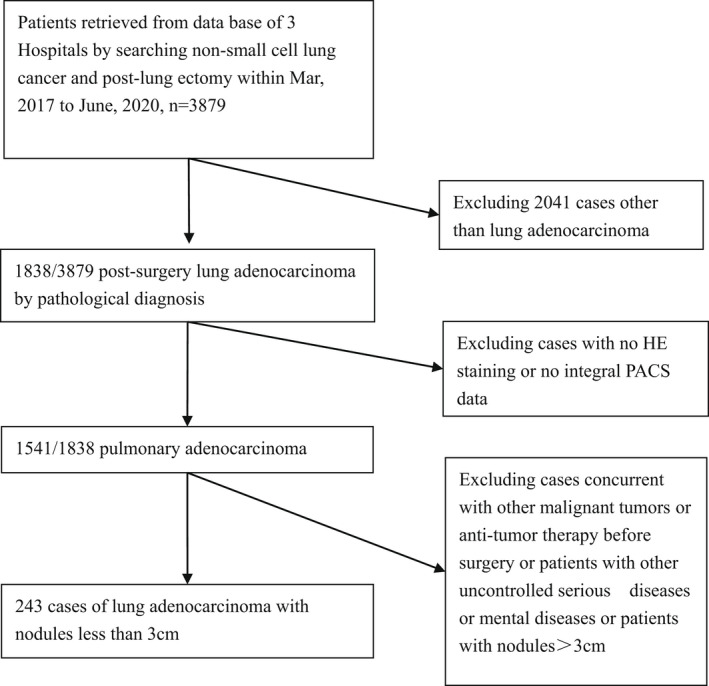
Flowchart of patient selection.

This study was approved by the Ethics Committee of the hospital (No. sjtkyll‐lx‐2020) prior to its commencement and exempted from informed patient consent. We retrospectively analyzed a total of 3879 patients with surgically resected NSCLC from three hospitals (Qingdao University Affiliated Hospital, Chinese People's Liberation Army General Hospital, and Beijing Shijitan Hospital Affiliated to Capital Medical University) between March 30, 2017 and May 30, 2020. Of the patients analyzed, 1838 with a postoperative pathological diagnosis of LAC were screened out, and the CT images were retrieved from the image archive and communication system (PACS). Patients who exhibited a solitary pulmonary nodule with a diameter of ≤3 cm on thin‐slice chest CT were further evaluated. Patients with the following criteria were excluded: (a) no pathological hematoxylin–eosin (HE)‐stained sections available, (b) no CT images available in PACS, (c) anticancer therapy before surgery, (d) other uncontrolled serious diseases or mental diseases, and (e) concurrent other malignant tumors. Finally, a total of 243 patients were enrolled in this study. The inclusion flowchart is shown in Figure 1.

### 2.2 CT image analysis

CT images of the whole lung were obtained using a 64‐slice CT scanner with a routine scan slice thickness of 2–5 mm. The cross‐sectional width of 2.0 mm and the reconstruction interval of 2.0 mm were adopted, and the thickness of the reconstructed cross‐section was 1–1.5 mm. All images were reviewed at a high resolution on a 20.8 inches monitor with a 2048 × 1560‐pixel gray‐scale, and the window setting displayed a standard lung (window width 1500 HU, window level 600 HU) and mediastinum (window width 350 HU, window level 50 HU). The CT images were reviewed by two chest radiologists with 10 or more years' experience to evaluate the density, size, and appearance of lung nodules. The inconsistent results were further analyzed and consensus was reached by multidisciplinary team, including two pulmonologists. Pulmonary nodules were divided into pure ground‐glass nodules (pGGNs), part‐solid nodules (PSNs), and solid nodules (SNs) according to CT features. Tumors were staged using the TNM staging system.^24^

### 2.3 Pathological analysis

HE stained sections of lung nodules surgically removed from patients were evaluated by two pathologists with no less than 10 years' experience. According to the classification for LAC jointly revised by IASLC/ATS/ERS, the nodules were divided into several types including lung adenocarcinoma in situ, minimally invasive adenocarcinoma (MIA), and lung invasive adenocarcinoma (IAC). Inconsistent analysis was clarified by consensus.

### 2.4 TLSs quantification

TLSs were assessed morphologically in MIA and IAC cases using HE staining, as reported previously.^25^, ^26^ TLSs were identified as (i) aggregates: clusters of lymphocytes with no distinct shape; (ii) primary follicles: lymphocyte clusters exhibiting dense, round shape with no germinal center formation, and (iii) secondary follicles: clusters of lymphocytes with germinal center formation.^10^ TLS maturity was further defined as Grade I (immature TLSs): tumor with only aggregates and no follicles; Grade II (semi‐mature TLSs): tumor with primary follicles with or without aggregates and without secondary follicles; and Grade III (mature TLSs): tumor with at least one secondary follicle. TLS density was quantified by the total number of TLSs identified in the tumor area, and the total number of TLSs that were in direct contact with tumor cells at the tumor edge (the number of TLSs per square millimeter tumor area).^27^

### 2.5 Statistical analysis

General clinical features of patients, including sex, age, smoking history, *EGFR* gene mutation tests, CT features, and pathological types were analyzed using descriptive methods. The correlation between the above‐mentioned clinical features and TLS occurrence was analyzed using binary logistic regression. The Wilcoxon rank‐sum test and further Poisson regression were used to evaluate the correlation between TLS density and clinical features. The TLS maturity was analyzed using the chi‐squared test and ordinal logistic regression. Statistical significance was set at *p* < 0.05. All data were analyzed using the STATA (version 16.0) software.

## 3 RESULTS

### 3.1 General clinical characteristics

In this study, 243 patients with early stage peripheral LAC, characterized by pulmonary nodules, were included. The general characteristics of the patients are summarized in Table 1. The profile of age, sex, smoking, and pathological types was in line with the characteristics of the clinical patient spectrum, except for higher frequency of *EGFR* mutations, as reported in the Asian population.^[^ ^28^, ^29^ ^]^

**TABLE 1:** General clinical characteristics

	*N*	
Sex		
Male	157	64.61%
Female	86	35.39%
Age	243	60.08 ± 1.96 × 0.55
Smoking		
Yes	40	16.46%
No	203	83.54%
EGFR mutation		
Yes	201	82.72%
No	42	17.28%
Pathological types		
AIS	4	1.65%
MIA	41	16.87%
IAC	198	81.48%
CT features		
pGGN	43	17.70%
PSN	112	46.09%
SN	88	36.21%
TLS cases	219	90.12%
TLS maturity		
Grade I	146	66.67%
Grade II	58	26.48%
Grade III	15	6.85%
Tumor stages		
I A1	72	29.6%
I A2	112	46.1%
I A3	25	10.3%
I B	26	10.7%
II B	8	3.3%

*Note*: Data presented are mean ± SD or *n* (%) of cases.

Abbreviations: AIS, adenocarcinoma in situ; IAC, invasive adenocarcinoma; MIA, minimally invasive adenocarcinoma; *N*, number of cases; pGGN, pure ground‐glass nodule; PSN, part‐solid nodule; SN, solid nodule.

### 3.2 TLSs characteristics

**FIGURE 2:**
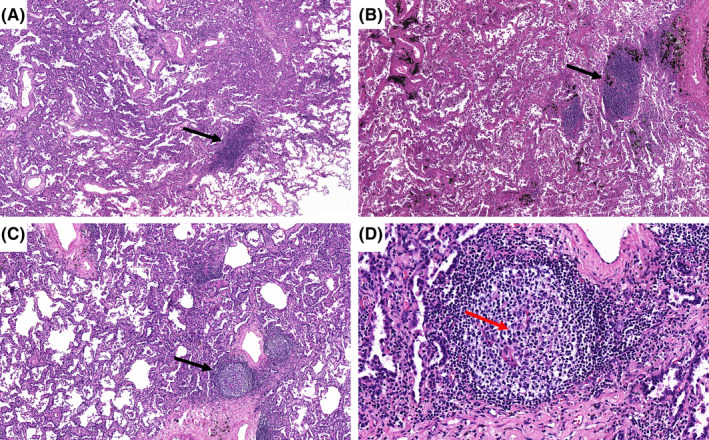
Histological appearance of intra‐tumoral TLSs. (A) Grade I TLSs: Aggregates are vague, ill‐defined clusters of lymphocytes (black arrow: HE×100). (B) Grade II TLSs: Primary follicles consist of dense, round, or oval shaped clusters of lymphocytes (black arrow: HE ×100). (C) Grade III TLSs: Secondary follicle are centered by a germinal center (black arrow: HE ×100). (D) Grade III TLSs: At high magnification, microscopic examination of a secondary follicle shows a pale area (germinal center‐red arrow), with a dense outer rim of lymphocytes (mantle zone) (HE ×400). HE, hematoxylin and eosin stain; TLSs, tertiary lymphoid structures. (This figure appears in color on the web).

**FIGURE 3:**
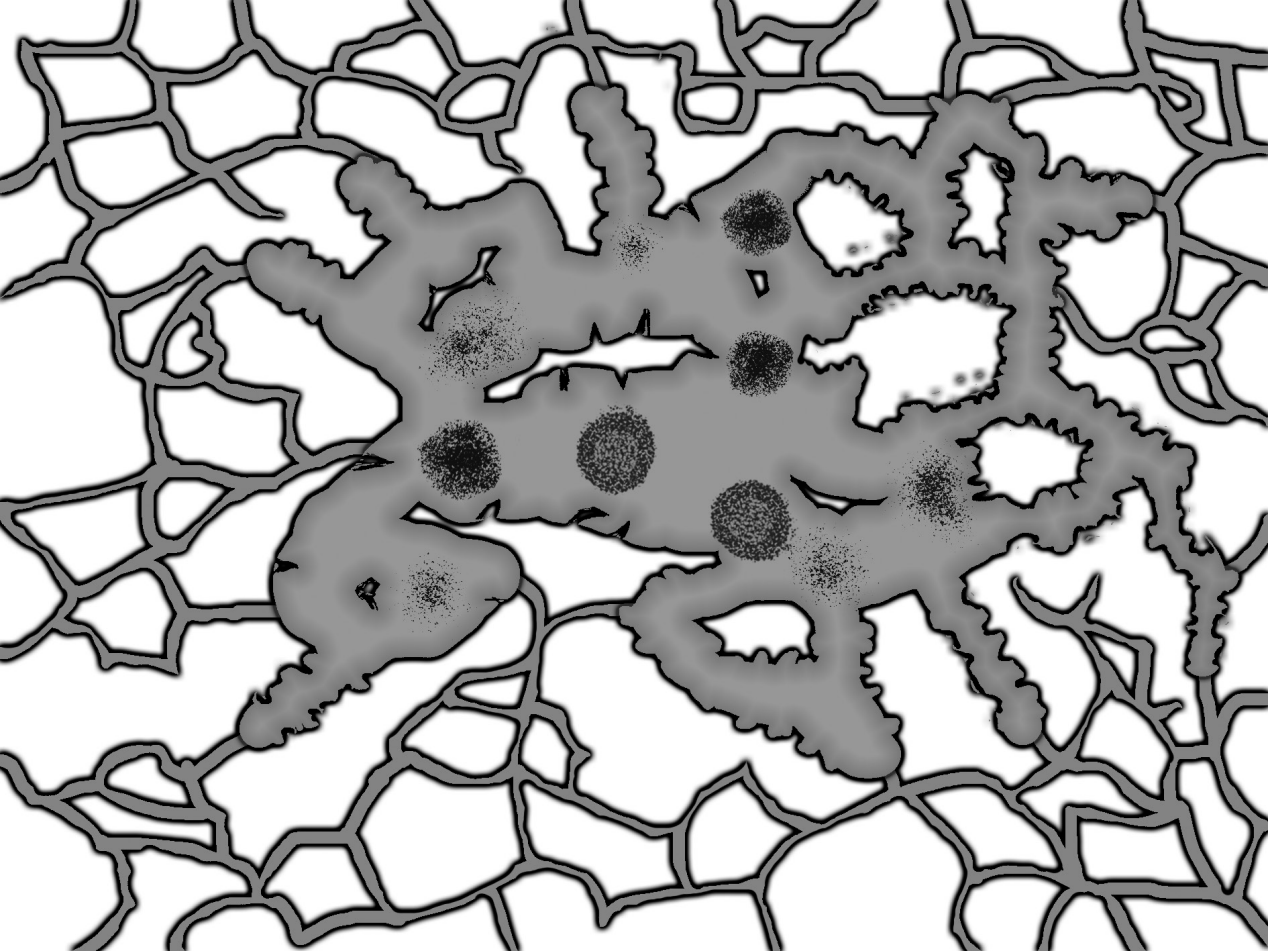
Mode pattern of coexistence of TLSs with different maturity in the same tumor. TLSs, tertiary lymphoid structures.

A total of 219 patients were confirmed to have TLSs by pathological analysis, accounting for 90.12% of the total patients (Table 1). Among the patients with TLSs, there were 15 with typical germinal center‐like structures (grade III TLSs), 58 with obvious lymphocyte clusters (grade II TLSs), and 146 with vague lymphocyte aggregation (grade I TLSs) (Figure 2). Moreover, the coexistence of different grades of TLSs and even all three grades could be found in the same tumor^30^ (Figure 3).

Clinical features were significantly correlated with the occurrence of TLSs as shown using the chi‐square test (Table 2). Further logistic regression showed that the probability of TLSs in pGGNs was significantly lower than that in PSNs (odds ratio [OR]: 0.29; 95% confidence interval [CI]: 0.104–0.812; *p* = 0.018). There was no significant difference between the PSNs and SNs.

**TABLE 2:** Clinical features and TLS occurrence

Clinical features	*p*
Age	0.153
Sex	0.502
Smoking	0.797
EGFR Mutation	0.216
CT Features	0.037
pGGN	OR:0.291; 95% CI: 0.104–0.812; *p* = 0.018
PSN	1
SN	OR:0.89; 95% CI: 0.31–2.557; *p* = 0.890
Pathological type	0.2
MIA	
IAC	
Tumor stages	0.173

*p* < 0.05 was considered significant.

Abbreviations: AIS, adenocarcinoma in situ; IAC, invasive adenocarcinoma; MIA, minimally invasive adenocarcinoma; pGGN, pure ground‐glass nodule; PSN, part‐solid nodule; SN, solid nodule.

### 3.3 TLS density and maturity

The relationship between general clinical features and TLS density/maturity is summarized in Table 3. No correlation was found between TLS density and sex, smoking, *EGFR* gene mutational status, and pathological types (*p* > 0.05), while age and CT features were found to be related to TLS density (both *p* < 0.05). Poisson regression analysis showed that TLS density was lower in patients over 60 years old than in those under 60 years old (incidence rate ratio [IRR]: 0.605; 95% CI: 0.4–0.92; *p* = 0.018). Furthermore, TLS density was significantly higher in PSNs and SNs than in pGGNs, with (IRR: 3.137; 95% CI: 1.35–7.27; *p* = 0.008) and (IRR: 2.44; 95% CI: 1.02–5.85; *p* = 0.046), respectively.

**TABLE 3:** TLS density and maturity in different groups

	TLS density(*p* value)	TLS maturity (Grade I II III) (*p* value)
Sex(F/M)	0.458 ± 0.09/0.351 ± 0.08(0.0545)	91–41‐87/55–17‐7 (0.360)
Age(60+/60‐)	0.335 ± 0.05/0.528 ± 0.132(0.0336)	78–36‐9/68–22‐6 (0.508)
Smoking(Y/N)	0.442 ± 0.16/0.418 ± 0.07(0.6179)	19–8‐6/123–50‐9 (0.051)
EGFR mutation(Y/N)	0.415 ± 0.06/0.434 ± 0.32(0.0598)	115–52‐13/31–6‐2 (0.218)
Pathological types		
MIA/AIS	0.443 ± 0.28/0.216 ± 0.23(0.6343)	27–5‐2/3–1‐0 (0.629)
IAC/AIS	0.420 ± 0.06/0.216 ± 0.23(0.2694)	116–52‐13/3–1‐0 (1.000)
MIA/IAC	0.443 ± 0.28/0.420 ± 0.06(0.1346)	27–5‐2/116–52‐13 (0.213)
CT features		
pGGN/mGGN	0.178 ± 0.06/0.522 ± 0.12(0.0001)	26–7‐1/67–28‐9 (0.397)
pGGN/SN	0.178 ± 0.06/0.388 ± 0.08(0.0003)	26–7‐1/53–23‐5 (0.587)
mGGN/SN	0.522 ± 0.12/0.388 ± 0.08(0.0526)	67–28‐9/53–23‐5 (0.813)
Tumor stages	0.183	0.026
IA1‐IA3	0.399 ± 0.03	130–45‐14
IB‐IIB	0.533 ± 0.14	15–13‐1

*p* < 0.05 was considered significant.

Abbreviations: AIS, adenocarcinoma in situ; MIA, minimally invasive adenocarcinoma; IAC, invasive adenocarcinoma; pGGN, pure ground‐glass nodule; PSN, part‐solid nodule; SN, solid nodule.

In the tests related to TLS maturity, no correlation was found with respect to age, sex, *EGFR* gene mutation, CT features, pathological types, or smoking, except for tumor stages by ordinal logistics regression. TLS maturity was higher in IB‐IIB stages than in IA1‐IA3 stages (*p =* 0.026).

**FIGURE 4:**
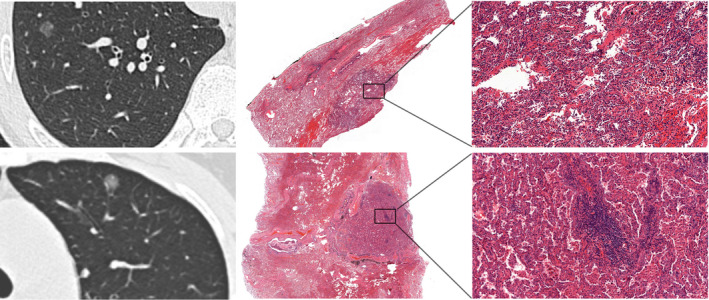
CT images and corresponding HE histologic findings in peripheral LAC characterized by pGGN. Top row: images in a 43‐year‐old man with LAC. CT manifestation is pGGN; a corresponding pathological manifestation has no obvious TLSs in tumor tissues. Bottom row: images in a 64‐year‐old woman with LAC. CT manifestation is pGGN, a corresponding pathological manifestation is grade I immature TLSs. CT, computed tomography; HE, hematoxylin and eosin stain; LAC, lung adenocarcinoma; pGGN, pure ground‐glass nodule; TLSs, tertiary lymphoid structures.

**FIGURE 5:**
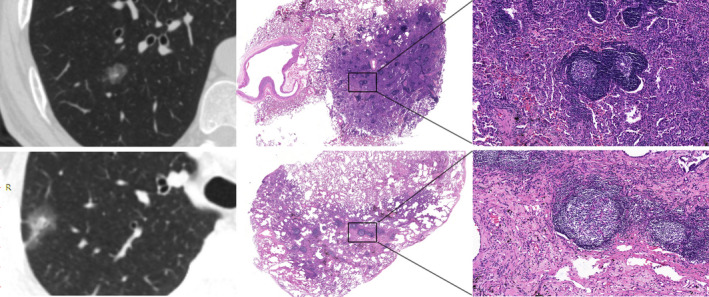
CT images and corresponding HE histologic findings in peripheral LAC characterized by PSNs. Top row: images in a 52‐year‐old man with LAC. CT shows PSNs and the corresponding pathological manifestation is grade III mature TLSs in tumor tissues. Bottom row: images in a 47‐year‐old woman with LAC. CT shows PSNs and the corresponding pathological manifestation is grade III mature TLSs in tumor tissues. CT, computed tomography; HE, hematoxylin and eosin stain; LAC, lung adenocarcinoma; PSN, part‐solid nodule; TLSs, tertiary lymphoid structures.

**FIGURE 6:**
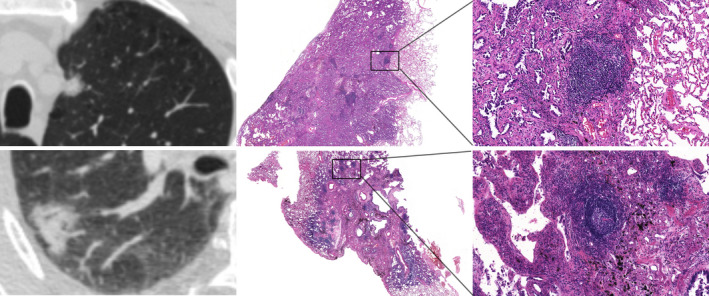
CT images and corresponding HE histologic findings in peripheral LAC characterized by SN. Top row: images in a 64‐year‐old man with LAC. CT shows SN and the corresponding pathological manifestation is grade II semi‐mature TLSs in tumor tissues. Bottom row: images in a 61‐year‐old woman with LAC. CT shows SN and the corresponding pathological manifestation is grade III mature TLSs in tumor tissues. CT, computed tomography; HE, hematoxylin and eosin stain; LAC, lung adenocarcinoma; SN, solid nodule; TLSs, tertiary lymphoid structures.

It is worth noting that although no correlation was found between TLS maturity and CT features, there was a tendency for less developed lymphoid structures in pGGNs (Figure 4) than in PSNs (Figure 5) and SNs (Figure 6).

## 4 DISCUSSION

In this study, we found that TLSs were ubiquitously present in LAC, with a structure of sparse lymphocytes aggregating to germinal center‐like structures that resembled typical lymphoid nodes, as it was shown in other tumors.^31^, ^32^ We first found that TLS occurrence was related to CT features and was lower in pGGNs than in PSNs and SNs. This result might be explained by the growth pattern of pGGNs, which consist of abnormally proliferating epithelial cells or well‐differentiated tumor cells growing in a scaly manner; and since tumor cells rarely breach the basement membrane of the alveolar septum, immune recruitment as well as TLS formation might be avoided.^33^

We also found that the density of TLSs was lower in the pGGNs. Although both the occurrence rate and TLS density were lower in pGGNs, this finding does not necessitate a poor prognosis as reported in other malignancies because the subjects in this study had mainly early stage tumors. In early LAC stages, tumor cells rarely break through the alveolar epithelium, and lymph node metastasis rarely develops in patients with only pGGNs.^34^ Therefore, it is doubtful that TLSs in pGGNs possess prognostic value.

When the tumor breaks through the basement membrane, the lung nodules would present more solid components and manifest as PSNs or SNs. Previous studies have suggested that the solid components in the lung nodules are caused by vascular proliferation, fibrosis, and alveolar cavity collapse, reflecting the invasive growth of LAC.^33^ In our study, it was found that the increase in the solid components in lung nodules was not only related to the increase in tumor cells, but also to the increase in the number of TLSs. However, in group comparison between PSNs and SNs, no difference was found in TLS numbers; thus, the transition from PSNs to SNs may be dominated by the increase in tumor cells and/or fiber components. These findings could help us to better understand the pathogenesis of early LAC stages.

It was also found that the number of TLSs were decreased with age, which may be related to a decline in the patient immune status. This finding might partly explain the compromised antitumor immune therapy responses in older patients.^35^, ^36^

In our study, tumor stages were found to be related to TLS maturity, which means more advanced TLS grades in higher tumor stages. This finding was in accordance with the better effect of immunotherapy observed in some higher‐stage tumors.^37^ Thus, TLS profile could be a predictor of immunotherapy irrespective of tumor stage. However, further study is needed because the five different tumor stages were combined into two groups, IA1‐IA3 and IB‐IIB, because of the limited number of cases in the IB and IIB stages.

We also found a tendency for more advanced TLSs in PSNs and SNs compared to pGGNs. Considering the significant roles of CT features in both TLS occurrence rate and density, their roles in TLS maturity warrant further study with larger number of cases to clarify if the tendency we observed in this study really exists.

It is noteworthy that the patients enrolled in this study were limited to early stage LAC, and the results obtained may not be generalizable to all LAC stages.

## 5 CONCLUSION

Our findings illuminate the existence of distinct profiles of TLSs in the early stages of LAC, showing their ubiquitous presence in all stages, but mainly in Grade I and Grade II. The occurrence rate and density of TLSs vary with CT features and are both lower in pGGNs, which might be explained by the growth pattern of pGGNs and thus might not necessitate a poor prognosis. The progression from pGGN to PSNs and SNs is accompanied with increased number of TLSs as well as other tumor components. Older age is also correlated with lower TLS density. TLS maturity correlates with tumor stage and might correlate with CT features. These findings provide important information on LAC tumor pathogenesis and management.

## CONFLICT OF INTEREST

The authors declare that they have no competing interest.

## ETHICAL APPROVAL

This study was approved by the Clinical Trial Ethics Committee of Beijing Shijitan Hospital (No. sjtky11‐1x‐2020[74]) before commencement. The requirement for written consent was waived.

## Data Availability

The datasets used and/or analyzed during the current study are available from the corresponding author on reasonable request.
